# Insulin gene polymorphisms in type 1 diabetes, Addison's disease and the polyglandular autoimmune syndrome type II

**DOI:** 10.1186/1471-2350-9-65

**Published:** 2008-07-11

**Authors:** Elizabeth Ramos-Lopez, Britta Lange, Heinrich Kahles, Holger S Willenberg, Gesine Meyer, Marissa Penna-Martinez, Nicole Reisch, Stefanie Hahner, Jürgen Seissler, Klaus Badenhoop

**Affiliations:** 1 Department of Internal Medicine I, Division of Endocrinology, Diabetes and Metabolism, University Hospital Frankfurt, Frankfurt am Main, Germany; 2 German Diabetes Research Institute, University Hospital Düsseldorf, Düsseldorf, Germany; 3 Department of Internal Medicine, Division of Endocrinology and Diabetes, University Hospital Munich, Munich, Germany; 4 Department of Internal Medicine I, Division of Endocrinology, University Hospital Würzburg, Würzburg, Germany

## Abstract

**Background:**

Polymorphisms within the insulin gene can influence insulin expression in the pancreas and especially in the thymus, where self-antigens are processed, shaping the T cell repertoire into selftolerance, a process that protects from β-cell autoimmunity.

**Methods:**

We investigated the role of the -2221Msp(C/T) and -23HphI(A/T) polymorphisms within the insulin gene in patients with a monoglandular autoimmune endocrine disease [patients with isolated type 1 diabetes (T1D, n = 317), Addison's disease (AD, n = 107) or Hashimoto's thyroiditis (HT, n = 61)], those with a polyglandular autoimmune syndrome type II (combination of T1D and/or AD with HT or GD, n = 62) as well as in healthy controls (HC, n = 275).

**Results:**

T1D patients carried significantly more often the homozygous genotype "*CC*" -2221Msp(C/T) and "*AA*" -23HphI(A/T) polymorphisms than the HC (78.5% vs. 66.2%, *p *= 0.0027 and 75.4% vs. 52.4%, *p *= 3.7 × 10^-8^, respectively).

The distribution of insulin gene polymorphisms did not show significant differences between patients with AD, HT, or APS-II and HC.

**Conclusion:**

We demonstrate that the allele "*C*" of the -2221Msp(C/T) and "*A*" -23HphI(A/T) insulin gene polymorphisms confer susceptibility to T1D but not to isolated AD, HT or as a part of the APS-II.

## Background

Type 1 diabetes (T1D) has a multifactorial pathogenesis, partly caused by environment but also by genetic predisposition. The association of the MHC class II genes with T1D is well known, but there are also non- MHC genes such as the insulin gene, that confer susceptibility to T1D [1].

The insulin gene is transcribed upon binding of the transcription factor Pur1 to the promoter element, known as the variable number of tandem repeat (VNTR) region, located 596 bp upstream of the insulin gene translation initiation site. The VNTR region comprises variable 14–15 bp tandem repeat sequences with the consensus 5'-ACAGGGGTGTGGGG-3', also named the insulin gene minisatellite. In Caucasians there are two main classes of the VNTR region minisatellites named class I and class III alleles. The VNT

R class I alleles, which are associated with increased susceptibility to T1D, comprise between 26 and 63 repeat units and are shorter than class III alleles (between 141 and 209 repeat units), associated with dominant protection. The class II alleles are very rare in Caucasians but common in Africans [2-5].

Polymorphisms within the insulin gene are in linkage disequilibrium with the VNTR region and can therefore be used as markers for these gene variants. The allele "*C*" of the -2221 MspI polymorphism of the insulin promoter region has been shown to be in strong linkage with the class I allele of the VNTR region and the variant "*T*" with the class III alleles [6,7]. Therefore this polymorphism can be used as a marker for this gene segment [8].

Recent studies of the insulin gene locus showed the -23HphI (A/T) and the +1140 (A/C) polymorphisms to confer the strongest susceptibility to T1D [4,9]. Furthermore polymorphisms of the insulin gene are associated with different regulation of insulin expression in target tissues such as the pancreas and especially in the thymus, where the expression of self-antigens shapes and maintains a self tolerant T cell repertoire [10,11].

T1D and Addison's disease (AD) may occur in the same patient to form a polyglandular syndrome type II (APS-II) [12]. Although the precise function of the VNTR is uncertain, it is conceivable that through its potential effect on the thymic expression level of insulin, the VNTR could affect the development of immune self tolerance not only to antigenic peptides derived from insulin but also to other endocrine targets: this could occur through local insulin signalling by affecting other genes' expression levels thereby leading to differences in thymic expression levels of thyroid or other endocrine antigens. Therefore the VNTR region could confer susceptibility not only to T1D but also to other autoimmune endocrine disease such as Addison's disease (AD), Hashimoto's thyroiditis (HT) or Graves' disease (GD) by causing an abnormal thymic tolerance [13].

Therefore we investigated the role of the the -2221Msp(C/T) and -23HphI(A/T) insulin gene polymorphisms in patients with isolated T1D, AD and HT and in patients with a APS-II [T1D and/or AD and at least one of the disorders HT or GD] as well as in healthy controls (HC).

## Methods

### Subjects

All patients were recruited from the endocrine outpatient clinics at the University Hospitals Frankfurt am Main, Freiburg, Würzburg, Munich and Düsseldorf (Germany).

Altogether 1248 individuals were genotyped for the -2221 (C/T) polymorphism within the promoter region of the insulin gene [485 with a monoglandular autoimmune disease, 317 patients with T1D (of these 213 with parents), 107 with AD and 61 with HT, 62 with APS-II and 275 HC].

#### T1D

T1D was diagnosed according to World Health Organization criteria. The median age of disease onset was 10 years (range 1–50 years). From the 213 T1D families, 17 offspring had T1D as a part of the APS-II. There was no evidence for MODY- or other rare forms of insulin-deficient diabetes, furthermore all were simplex cases.

Patients with isolated T1D may develop other autoimmune diseases in the future. Nevertheless, at the point of this study, they had the diagnosis of isolated T1D.

#### AD

Addison's disease was diagnosed by primary adrenal failure without evidence of tuberculosis or adrenoleukodystrophy. Adrenal autoantibodies were detected with indirect immunofluorescence on cryostat sections; these were confirmed to be directed against 21-hydroxylase in a subgroup of patients by radioimmunoassay. No neurological deficits could be detected. No patients with AD without T1D had antibodies against β-cell antigens (GAD-Ab or IA-2 Ab).

#### HT

The diagnosis of HT was established by positive thyreoglobulin (Tg) and/or thyroid peroxidase antibodies, reduced echogenicity on thyroid ultrasound, and normal or elevated thyrotropin (TSH) levels.

#### APS-II

Patients with an APS-II (n = 62) were diagnosed as a combination of T1D or AD and other of the above mentioned disorders.

Among the patients with APS-II, there were 22 patients with the diagnosis T1D and autoimmune thyroidtis (AIT) (15 with T1D and HT and 7 with T1D and GD), 33 with AD and AIT (23 with AD and HT and 10 with AD and GD) as well as 7 with T1D, AD and HT.

The diagnosis of GD rested on autoimmune hyperthyroidism with elevated TSH receptor autoantibodies and/or ophthalmopathy.

#### HC

Age- and sex-matched HC were volunteer blood-donors from the Red Cross Blood Transfusion Centre in Frankfurt am Main (Germany), staff personal or medical students from the University Hospital Frankfurt am Main (Germany) without family history of T1D, HT, GD or AD. Although the adrenal function of the controls was not formally assessed, they had normal thyroid function and were thyroid autoantibody negative and did not report clinical symptoms.

All individuals were of German origin and were inhabitants from the surrounding area of Frankfurt am Main (Germany).

The male: female ratio of patients with T1D, AD, HT, APS-II and HC was 1:0.91, 1:2.14, 1:4.56, 1:3.89 and 1:0.48, respectively. The median age of the patients with T1D, AD, HT, APS-II and HC was 30 (range 5–81), 57 (range 22–92), 52 (range 25–95), 46 (range 15–76) and 40.5 (range 23–83), respectively.

The study protocol was approved by the Ethics Committee of the University Hospital, Frankfurt am Main and written informed consent was obtained from all patients and controls.

### Genotype analysis

DNA was extracted from whole blood according to standard protocols and polymorphisms were typed according to the following protocols:

#### -2221MspI (C/T)

Patients with T1D, HT and APS-II and HC were studied for the insulin gene promotor polymorphism -2221 (C/T) using polymerase chain reaction (primers: 5'-ACCCCACTACACGCT GCTG-3' and 5'-CCCTTCAGAGACACCCCCA-3') followed by digestion with the restriction enzyme Msp I (New England Bio Labs, Beverly, MA, USA) and separated on 4% agarose gel.

DNA of patients suffering from AD as well as of 100 samples of the above mentioned (which were genotyped by restriction enzyme and chose at random to confirm the accuracy of the method) were analyzed by real-time PCR using ABI 7300 (Applied biosystems^®^). Primer and probe sequences are as follows: forward primer: 5'-TGAGTAGGAGTTTGCCAGTGTTG-3'; reverse primer: 5'-GCTGCTGGGATCCTGGAT-3'; probe for T allele 5'-VIC-TCAGCTCCCTGGCCGA-3' and probe for C allele 5'-FAM-AGCTCCCCGGCCGA-3'.

#### -23HphI (T/A)

All samples were genotyped by real time PCR by using C_1223317-Assay (Applied biosystems^®^). In order to check the accuracy of the method 100 DNA-samples – chosen at random- were genotyped according to a previously published [14].

### Statistical analysis

Patients and controls were compared using allele-wise and genotype-wise Chi^2 ^testing by using BiAS software (package 7.01, Epsilon, Weinheim, Germany) [15].

The transmission disequilibrium test (TDT) was used for the family analysis to detect preferential transmission of the alleles to affected offspring [16] as well as to analyze the interaction between HLA and the -2221 polymorphism using Haploview software version 3.2 available from the website [17].

In order to detect a statistical difference (p < 0.05) at an OR 1.8 (as seen for T1D) we calculated for the -2221Msp(C/T) SNP that 276 chromosomes (138 patients vs. 138 HC) and for -23HphI(A/T) 224 chromosomes (112 patients vs. 112 HC) are necessary to reach a power of 80%- using the software version 2.1.30 [18].

*P *values were corrected by the number of polymorphisms (n = 2). A corrected *p *(*p*c) < 0.05 was considered significant.

## Results

We found a significant difference in the genotype and allele distribution of both -2221Msp and -23HphI polymorphisms between patients with T1D and HC.

Thus T1D patients carried significantly more often the genotypes "*CC*" of the -2221MspI and "*AA*" of the -23HphI polymorphisms, respectively than the HC (78.5% vs. 66.2%, *p *= 0.0027 and 75.4% vs. 52.4%, *p *= 3.7 × 10^-8^, respectively, table 1 and 2).

**Table 1:** Distribution of genotypes of the -2221 (C/T) insulin polymorphism in German patients with autoimmune endocrinopathies and healthy controls

		**Genotype**									
						
		**CC**		**CT**		**TT**			**Allele**		
							
**Group**	**n**	**n**	**%**	**n**	**%**	**n**	**%**	p*	**C%**	**T%**	p*
**HC**	275	182	66.2	85	30.9	8	2.9		81.6	18.4	
**T1D**	317	249	78.5	64	20.2	4	1.3	0.0027	88.6	11.4	8.9 × 10^-4^
**HT**	61	36	59.0	22	36.1	3	4.9	0.4898	77.0	23.0	0.2998
**AD**	107	73	68.2	25	23.4	9	8.4	0.0331^†^	79.9	20.1	0.4738
											
**APS-II**	62	45	72.6	16	25.8	1	1.6	0.5870	85.5	14.5	0.3763

**T1D + AIT**	22	18	81.8	4	18.2	0	0	0.2898	90.9	9.1	0.2898
**T1D + HT**	15	13	86.7	2	13.3	0	0	0.2479	93.3	6.7	0.1653
**T1D + GD**	7	5	71.4	2	28.6	0	0	0.8851	85.7	14.3	0.9682
											
**AD + AIT**	33	22	66.7	10	30.3	1	3.0	0.9968	81.8	18.2	0.8948
**AD + HT**	23	15	65.2	8	34.8	0	0	0.6790	82.6	17.4	0.9723
**AD + GD**	10	7	70.0	2	20.0	1	10	0.3802	80.0	20.0	0.9138
											
**T1D + AD + HT**	7	5	71.4	2	28.6	0	0.0	0.8851	85.7	14.3	0.9682

P values are given uncorrected

*compared to healthy controls

^†^Not significant after correction

**Table 2:** Distribution of genotypes of the -23HphI (A/T) insulin polymorphism in German patients with autoimmune endocrinopathies and healthy controls

		**Genotype**									
						
		**AA**		**AT**		**TT**			**Allele**		
							
**Group**	**n**	**n**	**%**	**n**	**%**	**n**	**%**	p*	**A%**	**T%**	p*
**HC**	275	144	52.4	118	42.9	13	4.7		73.8	26.2	
**T1D**	317	239	75.4	70	22.1	8	2.5	3.7 × 10^-8^	86.4	13.6	6.6 × 10^-8^
**HT**	61	32	52.5	29	47.5	0	0	0.2092	76.2	23.8	0.6623
**AD**	107	54	50.5	46	43.0	7	6.5	0.7641	72.0	28.0	0.6675
											
**APS-II**	62	35	56.5	23	37.1	4	6.5	0.6485	75.0	25.0	0.8746

**T1D + AIT**	22	16	72.7	5	22.7	1	4.5	0.1675	84.1	15.9	0.1848
**T1D + HT**	15	11	73.3	3	20.0	1	6.7	0.2159	83.3	16.7	0.3436
**T1D + GD**	7	5	71.4	2	28.6	0	0	0.5628	85.7	14.3	0.4873
											
**AD + AIT**	33	17	51.5	13	39.4	3	9.1	0.5572	71.2	28.8	0.7597
**AD + HT**	23	13	56.5	8	34.8	2	8.7	0.5870	73.9	26.1	0.8724
**AD + GD**	10	4	40.0	5	50.0	1	10	0.6242	65	35	0.5353
											
**T1D + AD + HT**	7	2	28.6	5	71.4		0	0.3087	64.3	73.8	0.6227

P values are given uncorrected

*compared to healthy controls

^†^Not significant after correction

Heterozygous parents in T1D families transmitted significantly more often the allele "*C*" and "*A*" of the -2221MspI and -23HphI polymorphisms to their affected offspring (73.8% vs. 26.2 %, *p *= 9.0 × 10^-8 ^and 3.3 × 10^-8^, respectively; data not shown), matching our findings from the case-control analysis.

The -23HphI polymorphism showed the strongest association to T1D in both T1D-family and case-control analysis.

Neither genotype frequencies nor allele wise comparison differed between patients with HT, AD, APS-II and HC (table 1 and 2). No interaction was found when AD patients were stratified according to the HLA risk (data not shown).

## Discussion

In the present study, we demonstrate the allele "*C*" of the -2221MspI and "*A*" of the -23HphI polymorphisms within the insulin region to confer susceptibility to T1D in German patients. Therefore our data are in line with previous reports, which found that the -2221 MspI SNP and the -23HphI SNP are associated with T1D, where the -2221 MspI SNP was only part of a susceptibility insulin gene haplotype and the -23HphI SNP was found with the strongest association to T1D [4,9].

Other studies found no evidence of excessive transmission of the -23HphI polymorphism (equivalent to VNTR class I) in patients with GD [19]. Also no association was demonstrated of INS VNTR in patients with celiac disease and AD from a Basque population [20]. Thus our present study supports those findings suggesting that neither the -2221 MspI nor the -23HphI polymorphisms are associated with isolated AD, HT or with GD as part of the APS-II.

Class III alleles of the insulin VNTR region are associated with substantially higher insulin mRNA levels than VNTR class I in the thymus presumably, causing a more efficient deletion of insulin autoreactive T cells [13], explaining their protective effect on T1D. On the other hand the "*C*" allele of -2221 MspI has been associated with a decreased insulin expression in the thymus, leading to less efficient deletion of autoreactive insulin-specific T-cells [10]. This may increase the risk to develop T1D. Likewise the protective effect of the "*T*" [2] allele could lead to higher level of insulin transcription.

This association appears to be confined to patients with T1D only and larger numbers are needed to rule out an effect in patients with other immune components of T1D associated with the APS-II. So far this locus may help to differentiate the susceptibility to isolated T1D in comparison to T1D predisposition loci that are shared with endocrine autoimmune disorders.

## Conclusion

We demonstrate the allele "*C*" of the -2221MspI and "*A*" of the -23HphI polymorphisms within the insulin region confer susceptibility to T1D but not to isolated AD, HT or as a part of the APS-II.

## Abbreviations

T1D: Type 1 diabetes; VNTR: variable number of tandem repeat; AD: Addison's disease; HT: Hashimoto's thyroiditis (HT); APS-II: Polyglandular autoimmune syndrome type II; GD: Graves' disease; HC: Healthy controls; TDT: Transmission disequilibrium test; HLA: Human Leukocyte Antigen.

## Competing interests

The authors declare that they have no competing interests.

## Authors' contributions

ER–L and BL carried out the genotyping of the patients and controls. MP–M carried out the re-genotyping of the patients and controls to confirm the accuracy of the method. HK participated in the statistical analysis GM, HSW, NR, SH and JS made the diagnosis and collaborated in collection of samples. HK carried out statistical analysis. ER–L and KB conceptualized and designed the study.

## Pre-publication history

The pre-publication history for this paper can be accessed here:


